# The Macaque Gut Microbiome in Health, Lentiviral Infection, and Chronic Enterocolitis

**DOI:** 10.1371/journal.ppat.0040020

**Published:** 2008-02-01

**Authors:** Philip McKenna, Christian Hoffmann, Nana Minkah, Pyone Pyone Aye, Andrew Lackner, Zongzhi Liu, Catherine A Lozupone, Micah Hamady, Rob Knight, Frederic D Bushman

**Affiliations:** 1 Department of Microbiology, University of Pennsylvania School of Medicine, Philadelphia, Pennsylvania, United States of America; 2 Tulane National Primate Research Center, Tulane University Health Science Center, Covington, Louisiana, United States of America; 3 Department of Chemistry and Biochemistry, University of Colorado at Boulder, Boulder, Colorado, United States of America; 4 Department of Molecular, Cellular and Developmental Biology, University of Colorado at Boulder, Boulder, Colorado, United States of America; 5 Department of Computer Science, University of Colorado at Boulder, Boulder, Colorado, United States of America; Harvard Medical School, United States of America

## Abstract

The vertebrate gut harbors a vast community of bacterial mutualists, the composition of which is modulated by the host immune system. Many gastrointestinal (GI) diseases are expected to be associated with disruptions of host-bacterial interactions, but relatively few comprehensive studies have been reported. We have used the rhesus macaque model to investigate forces shaping GI bacterial communities. We used DNA bar coding and pyrosequencing to characterize 141,000 sequences of 16S rRNA genes obtained from 100 uncultured GI bacterial samples, allowing quantitative analysis of community composition in health and disease. Microbial communities of macaques were distinct from those of mice and humans in both abundance and types of taxa present. The macaque communities differed among samples from intestinal mucosa, colonic contents, and stool, paralleling studies of humans. Communities also differed among animals, over time within individual animals, and between males and females. To investigate changes associated with disease, samples of colonic contents taken at necropsy were compared between healthy animals and animals with colitis and undergoing antibiotic therapy. Communities from diseased and healthy animals also differed significantly in composition. This work provides comprehensive data and improved methods for studying the role of commensal microbiota in macaque models of GI diseases and provides a model for the large-scale screening of the human gut microbiome.

## Author Summary

Bacterial mutualists within the gastrointestinal tract aid digestion, promote development of the gut immune system, and provide competitive barriers to pathogen invasion. The host, in return, provides bacteria with safe housing and food during lean times. The composition of the gut microbiota is controlled in part by the host immune system. In a variety of disease states, immune function can be altered, and gut morbidity is often associated, leading to the hypothesis that alterations in the GI microbiota may contribute to disease. In this study, the gut microbiota was characterized in 100 samples from rhesus macaques using pyrosequencing, which allowed 141,000 sequences from 16S rRNA genes to be generated and analyzed. Healthy animals were compared to animals with gut disorders, induced, for example by advanced simian AIDS. Many factors contributed to changes in the microbiota, including the sex of the animal of origin. Animals with chronic colitis showed differences in composition of the GI microbiota compared to healthy animals, providing an association between altered microbiota and disease.

## Introduction

The human intestine is home to some 100 trillion microorganisms of at least 400 species. The density of bacterial cells in the colon has been estimated at 10^11^ to 10^12^ per ml, which makes it one of the most densely populated microbial habitats known [1,2]. The number of unique genes in the microbial pool is estimated to outnumber the genes in the human nuclear genome by two orders of magnitude [1,2], and these genes contribute many essential metabolic functions to the host. The great majority of gut bacterial species have not been cultured outside the human host and are known only by fragments of their DNA sequences. A few pioneering reports have begun to survey the intestinal microbiota of humans and mice using DNA sequencing of uncultured communities [1,3,4] or using microarray-based methods [5,6]. It is widely expected that human disease states will be linked to characteristic transitions in the intestinal microbiota, and connections have been proposed between GI bacterial communities and obesity [7,8] and Crohn's disease [9,10], but studies in this area are just beginning. Here we report characterization of GI microbial communities in rhesus macaques and their alteration accompanying colitis associated with SIV infection or in animals with chronic enterocolitis.

The mammalian GI tract is a major locus of immune tissues responsible for blocking invasion by pathogens, and more recently, these tissues have been implicated in normal homeostasis of the gut microbiota as well. For example, B-cells of the gut associated lymphoid tissues (GALT) synthesize IgA, which is secreted in large amounts into the lumen of the gut, and mice genetically incapable of normal IgA synthesis have abnormally large proportions of anaerobes in the small intestine [11,12]. Secreted antibacterial peptides have also been implicated in regulating the composition of the gut microbiota [13,14]. Effects of host genotype are also documented by the finding that genetically obese mice have detectably different gut microbiota compared to wild-type controls [8].

HIV infection causes rapid and massive destruction of GALT [15–20], and HIV infection is also frequently associated with gastrointestinal disorders, many of which are of unexplained etiology [21]. Destruction of GALT and gastrointestinal disorders are also a well-characterized consequence of simian immunodeficiency virus (SIV) infection in macaques [15,16,22–24]. A role for the GI microbiota in AIDS disease progression has recently been suggested–bacterial antigens are proposed to pass through the damaged GI mucosa and promote immune activation, which in turn promotes viral replication and disease progression [20].

Chronic enterocolitis is fairly common in rhesus macaques even in the absence of SIV infection or other known infectious or parasitic agents. Analysis of the clinical course and histopathology of idiopathic chronic enterocolitis shows many parallels with human inflammatory bowel disease (IBD), and indeed the macaque disease has been studied as a model for the human disorder [24–26]. Evidence of proinflammatory dysfunction of the IL6-JAK-STAT3-SOCS3 pathway has been reported [24]. A role for the gut microbiota in human IBD has long been suspected, and several studies have profiled uncultured GI bacteria from healthy and diseased patients (e. g. [9,27]). Such studies have not yet yielded a clear-cut picture of the relationship of GI microbiota to pathogenesis, though a reduction in microbial diversity has been proposed. Studies of the macaque disease have suggested that several GI microbes may be slightly more common in macaques with an IBD-like disease, but the macaque GI communities have not been comprehensively analyzed [20,25].

Here we characterize the macaque GI microbial communities and compare community composition in health and GI disease. To profile the bacterial taxa present, we purified bacterial DNA from samples of intestinal contents, amplified segments of the 16S rRNA gene, determined the sequences using massively parallel pyrosequencing, then used these data to identify and quantify the types of bacteria present [28]. The approach used here was based on extensive reconstruction studies [29], which showed that known clustering of microbial communities could be recaptured using 16S rRNA gene sequences of lengths generated by pyrosequencing using technology commercialized by 454 Life Sciences [30]. These preliminary bioinformatic studies also disclosed that some short segments of the 16S rRNA gene sequence were especially useful for phylogenetic reconstruction, allowing optimized primers to be chosen for the study reported here.

We found that the macaque microbiota was distinct from other vertebrates studied previously. Even in healthy animals the taxa present in the gut microbiota differed between individuals and changed substantially within individuals over time. Unexpectedly, communities from males and females also differed. Distinctive GI microbial communities were also obtained in samples of colonic contents taken at necropsy from animals with GI disease. Most of these animals were also treated with antibiotics to ameliorate their symptoms, so our analysis models human cases of colitis accompanied by antibiotic therapy. These data indicate that colitis and its treatment are associated with transitions in GI microbiota in the macaque, providing a model that may be useful in understanding the human GI microbiota in health and disease.

## Results

### Monitoring Macaque Intestinal Microbiota

We surveyed a range of sample types and disease states for possible effects on the macaque GI microbiota. We analyzed a total of 100 samples, including healthy animals, SIV infected animals, and animals with chronic enterocolitis. For the colitic samples, some of the animals were SIV infected and had colitis as a result of simian AIDS, while others were colitic but not SIV infected. Sample types included colonic contents collected at necropsy, bacterial communities adhered to biopsy specimens from the upper and lower GI mucosa (jejunum and colon respectively), and stool (Table 1; detailed data for each animal is in Table S1).

**Table 1:**
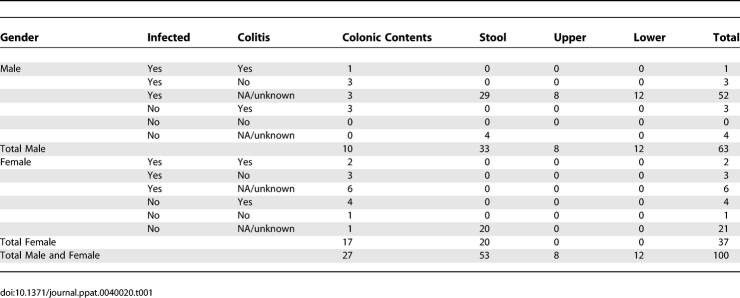
Samples of GI Microbiota Analyzed in This Study

**Figure 1:**
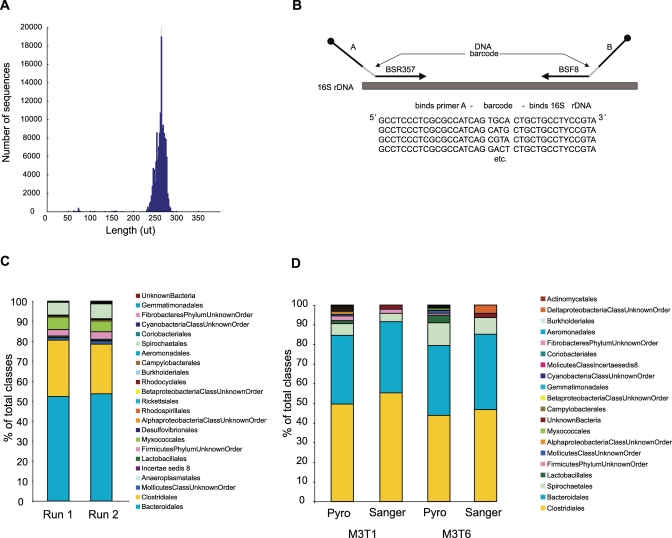
Use of DNA Bar-coding and Pyrosequencing to Analyze Uncultured Bacterial Communities (A) Length distribution of the pyrosequence reads used in this study. The median length was 264 nt. (B) The DNA bar-coding strategy. PCR primers are indicated by the arrows, DNA 5′ ends are shown as balls. Each primer contains a region complementary to the 454 sequencing primers (either A or B) and the 16S rRNA gene DNA (either BSR357 or BSF8) separated by a unique 4 base bar code (bold). (C) Reproducibility of the pyrosequencing method. DNA from a single specimen was analyzed by pyrosequencing at two different centers. Bacterial orders are indicated by the color code. (D) Comparison of results with pyrosequencing and conventional Sanger sequencing. Bacterial orders are indicated by the color code. Numbers of sequences are as follows: M3T1 pyrosequence, 1382; M3T1 Sanger sequence, 47; M3T6 pyrosequence, 1360; M3T6 Sanger sequence, 47.

DNA was isolated from all 100 samples and amplified by PCR using primers BSR357-A and BSF8-B, which anneal to conserved regions of bacterial 16S rRNA gene. All sequence reads extended from the BSR357-A primer. The median read length was 264 nt (Figure 1A). These primers were chosen based on a series of simulations carried out to investigate the optimal region of the 16S rRNA gene to query using the short sequence reads expected from pyrosequencing. Use of a moderately conserved region yielded relatively stable phylogenetic placements, though at the expense of reduced ability to discriminate low-level taxa. Biased amplification of 16S rRNA gene sequences from mixed bacterial populations can lead to distortions in abundance estimates, but these are typically in the range of only a few fold [6,31–33]. To facilitate comparison among samples, only a single region of the 16S rRNA gene was amplified, and uni-directional reads were used for the analysis, so that any biases introduced during amplification are common among all samples.

The primers for the 16S rRNA gene sequences were each marked with a unique DNA bar code by including distinctive 4 base sequences in the primers between the 16S rRNA gene complementary region and the binding sites for the pyrosequencing primers (Figure 1B). This allowed the PCR products from many samples to be sequenced using the 454 Life Sciences [30] technology, then indexed afterwards. After removal of low quality sequences, a total of 140,356 useable sequence reads were obtained. All bar codes were well populated, with an average of 1404 sequences per community tested. The error rate for the bar coding procedure could be estimated by cataloging all those sequence reads with bar codes that were not among those used for labeling. The analysis indicated that only 0.01% of sequences were likely to be miscataloged due to errors parsing the bar codes.

One DNA sample was sequenced twice to assess reproducibility. To determine the bacterial taxa present, the 16S rRNA gene sequences were aligned using NAST and GREENGENES and then inserted into pre-established phylogenetic trees of full length 16S rRNA gene sequences [34,35] using ARB. Over all the sequences analyzed in this study, 99.94% sequences aligned with previously determined 16S rRNA gene sequences (80 total sequences failed to align). The bacterial taxa in each sample were then tabulated. Comparison of the two independent sequencing experiments showed excellent reproducibility of the phylogenetic assignments (Figure 1C).

Ninety-four near-full length macaque bacterial 16S rRNA gene sequences from two communities were also determined by conventional Sanger sequencing to provide a check on the pyrosequencing data (Figure 1D; Table S2). As can be seen from Figure 1D, the major types and relative numbers of taxa were closely similar in the Sanger and pyrosequencing analysis for each sample, indicating that the pyrosequencing data yielded an accurate reflection of the species detected by conventional sequencing, though the minor taxa detected by pyrosequencing could not be detected in the Sanger reads due to the lower number of the latter.

### Microbial Diversity in the Macaque Intestinal Microbiota

**Figure 2:**
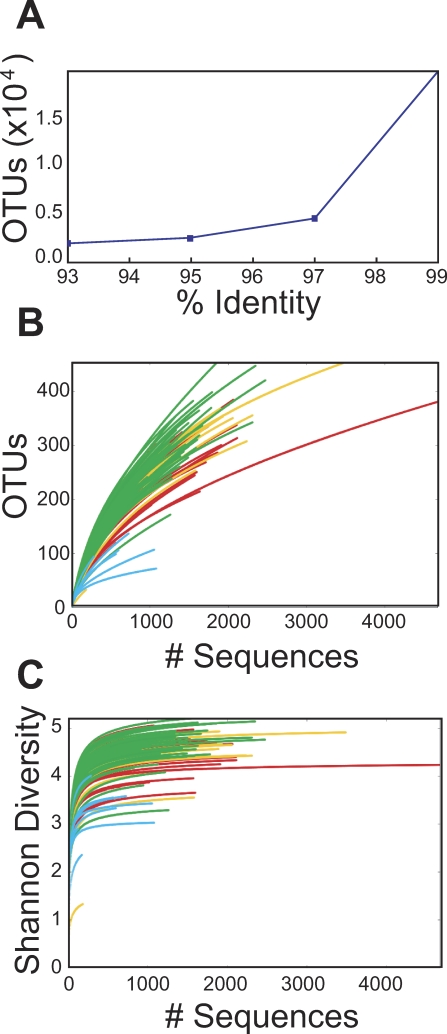
Diversity of the Macaque GI Microbiota (A) The numbers of operational taxonomic units (OTUs) present in the collection of pyrosequence reads was analyzed by condensing sequences at several percent identity thresholds. The *x*-axis shows the percent identity, the *y*-axis the number of OTUs detected. (B) Collectors curves analysis of the completeness of sampling. Repeated samples of OTU subsets were used to evaluate whether further sampling would likely yield additional taxa (rarefaction analysis), as indicated by whether the curve has reached a plateau value. The *y*-axis indicates the number of OTUs detected, the *x*-axis the number of taxa in the sequence subset analyzed. The color codes are as follows: green, stool samples; yellow, colonic contents; red, lower GI mucosal surface; blue, upper GI mucosal surface. (C) Rarefaction curves to estimate the diversity of taxa present in individual samples, using the Shannon Index. Color code as in (B). The upper GI mucosal samples were significantly less diverse than the other groups (*p* < 0.004 for pairwise comparisons of upper gut samples to each of the other three; Mann-Whitney comparison of means).

We first investigated the bacterial diversity present in our 16S rRNA gene sequence data. Sequence reads were aligned using NAST and compiled in OTUPicker. When sequences were condensed under conditions demanding 99% identity, about 20,000 different operational taxonomic units (OTUs; groups defined by pairwise sequence identity) were found (Figure 2A). When OTUs were defined using a threshold of 97% identity or greater, a criteria that in previous studies was judged to match roughly the species level [36,37], about 5,000 OTUs were identified. Errors introduced during pyrosequencing may influence this value, but effects are expected to be small (discussed further in the Methods section). In an effort to determine whether all the OTUs present in the data set had been recovered in the pyrosequencing study, a rarefaction analysis was carried out (Figure 2B). Increasingly large random subsets of the initial group of OTUs were analyzed for OTU number, and the totals plotted. If all the OTUs in the sample had been sequenced multiple times, a stable estimate would be reached at OTU values less than the number present in the full data set. As can be seen in Figure 2B, the estimates are still climbing even at the highest numbers of OTUs analyzed, indicating that substantial numbers of unseen OTUs exist in the samples and would only be detected after determining larger numbers of sequences.

In an attempt to estimate the total number of OTUs in each data set, the Chao 1 estimator was used, which uses frequency of isolation information to estimate the number of unseen OTUs present in the original sample. For most of the samples, the rarefaction curves on the Chao 1 estimates did not reached a stable value, indicating that the true numbers of OTUs in the samples are larger even than the Chao 1 estimates (53–1185 OTUs per sample; 97% identity criteria). Overall the richness of the bacterial taxa in the macaque GI microbiota was very high.

A comparison of the estimated diversity in all 100 samples was carried out by computing the Shannon Diversity Index from the OTU data for each sample (Figure 2C). To investigate the relative diversity at different anatomical sites, the 100 communities were grouped by sample type and their relative diversity compared. Rarefaction analysis indicated that most of the Shannon Diversity estimates had reached stable values. Separating the communities by sample type indicated that the upper GI mucosal samples from the jejunum were notably less diverse than the other groups.

### Comparison of the Macaque, Human, and Mouse GI Microbiota

**Figure 3:**
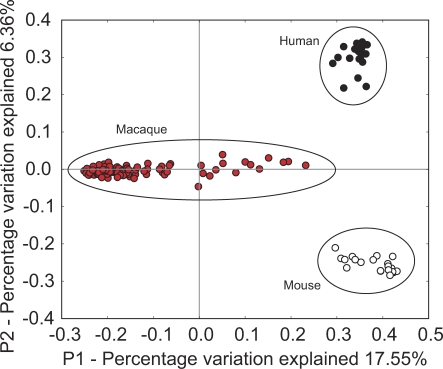
Comparison of the Macaque GI Microbiota to That of Mice and Humans The plot was generated using unweighted UniFrac. Mouse and human sequences were trimmed to match the macaque pyrosequence reads in length (264 nt) and location within the 16S rRNA gene. The differences among communities from the different vertebrates was significant at *p* < 0.001 (t-test with permutation).

A comparison of the macaque GI microbiota to that of humans [4] and mice [8] is shown in Figure 3. To compare the global compositions of microbial communities, we used UniFrac [38–40], which measures the similarity among bacterial communities based on phylogenetic distances. To carry out a UniFrac analysis, we used the augmented ARB database described above. To compare two communities using UniFrac, sequences from the two communities are marked on a common phylogenetic tree that contains all the sequences from the communities to be analyzed, and the fraction of the branch length on the tree unique to each community is then computed. This procedure provides a measure of the similarity between the two communities in terms of the total amount of evolutionary history that separates the sequences in the two communities. UniFrac assigns only a small difference to changes in representation of closely-related taxa, but larger value for changes in representation of more distant taxa, in contrast to OTU-based methods that assume that all taxa are equally distinct. To compare multiple communities, all the pair-wise distances between communities were computed, then Principal Coordinate Analysis (PCoA) was used to cluster the communities along axes of maximal variance (Figure 3).

To compare human and mouse samples to the macaque pyrosequencing data, sequence reads determined by the Sanger method from human and mouse were first truncated to match the length and position of the macaque 16S rRNA gene sequences. The UniFrac comparison showed strong clustering by species of origin. Similar separation by species was obtained when pyrosequencing data was used for both the rhesus and murine samples (unpublished data). For the human and macaque samples, the communities clustered by species of origin even though samples from diverse anatomical sites were included for each species.

**Figure 4:**
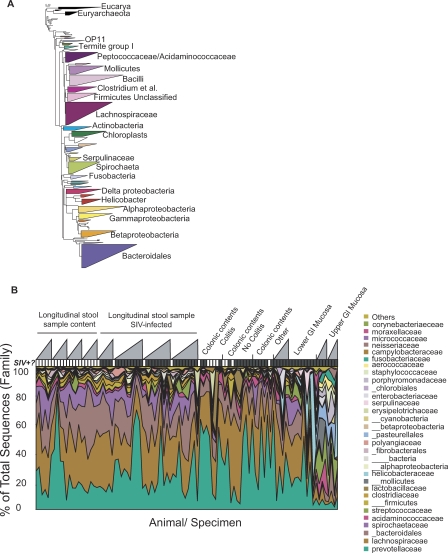
Bacteria Composing the Macaque GI Microbiome (A) Bacterial taxa identified from pyrosequencing data after alignment with the ARB 16S rRNA gene database. The size of each triangle indicates the relative number of OTUs within each taxa (100% identify threshold). (B) Summary of the bacterial taxa present in each gut community sampled, indicating the individual and temporal variation in the macaque GI microbiota. Each sample analyzed is indicated along the *x*-axis, the *y*-axis indicates the percent of the community comprised by each type of bacteria. A key to the bacterial taxa is listed at the right. Taxa corresponding to bacterial phyla are indicated with the triple underscore before the name, classes by a double underscore, orders by single underscores, and families by no underscore. Specific values for each community, along with clinical parameters for each monkey studied are summarized in Table S1.

The taxonomic groups from GI communities of each species were then compared. The bacterial taxa detected are summarized in Figure 4A. The most prominent bacterial classes were *Clostridia* (Phylum *Firmicutes*), *Bacteroidetes* (Phylum *Bacteroidetes*) and *Spirochaetes* (Phylum *Spirochaetes*). Present in lesser amounts are *Bacilli* and *Molicutes* (Phylum *Firmicutes*), *Alpha*, *Beta*, *Gamma*, and *Epsilon Proteobacteria*, and a collection of additional classes. Several of the minor classes were found repeatedly in specific individual macaques (e. g. *Fibrobacteres*, *Gemmatimonadetes*, *Deferribacteres*). All of the animals showed variation over time, in both the classes detected and in their relative abundance. Many of the bacterial taxa identified were not previously known to be present in the macaque intestinal microbiota.

The predominance of the phyla *Firmicutes* and *Bacteroidetes* were similar in all three vertebrates, and several lower-abundance phyla also overlapped. For example, *Proteobacteria* and *Actinobacteria* were found in both macaques and humans. *Verrucomicrobia* were detected in humans but were rare macaques.

A distinctive feature of the macaques was the density of Phyla *Spirochaetes,* particularly members of the genus *Treponema*, which were present in abundance in macaques (Figure 4) but mostly absent in the samples from in mice and humans. The abundance of flagellated *Helicobater* (*EpsilonProteobacteria*) has previously been noted [41], and *Spirochaetes* have been identified in the gut microbiota of many vertebrates including humans and non-human primates [42,43]. However, the abundance of *Treponema* in macaques was unexpected and far greater than in human.

In humans, within the Class *Bacteroidetes*, members of the genus *Bacteroides* have been reported to be a major and functionally significant component of the human intestinal microbiota [4,44,45], but of the 94 near full length 16S rRNA gene macaque sequences, only one was genus *Bacteroides*. More common were genus *Prevotella* (16/94 sequences), which is also common in humans, and *Rikenella* (18/94 sequences), which is rare or absent in humans [4]. These proportions of *Bacteroidaceae* and *Prevotellaceae* were similar in the shorter pyrosequencing reads.

In macaques, comparison of microbial communities among animals showed considerable variation among individuals, both in the relative abundance of the major taxonomic groups and in the presence of minor groups (Figure 4B). For some animals, longitudinal samples were available, showing that the composition of the GI microbiota was quite dynamic over the period of sampling.

### Distinctive Microbial Communities Associated with Different Anatomical Sites

**Figure 5:**
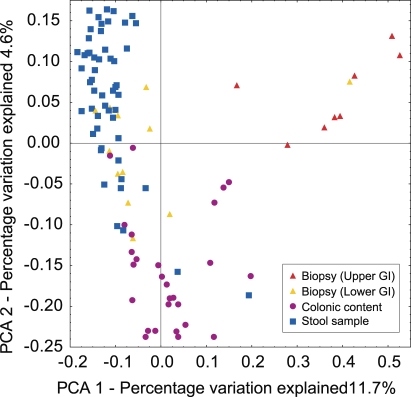
Distinctive GI Microbiota in Samples from Communities from Different Anatomical Sites Unweighted UniFrac was used in the comparison. The types of samples studied are indicated by the key at lower right.

Figure 5 shows a UniFrac clustering diagram comparing the communities from different anatomical sites. Possible clustering by sample type on the first two principal coordinates was assessed using a t-test to compare the within-group and between-group distances, then 1,000 label permutations were used to assess significance. Clustering for all four sample types was found to be significant at the *p* < 0.01 level.

The samples from the upper GI mucosa formed a distinct cluster to the upper right of the diagram, indicating unique composition. Analysis of the taxa present indicates that the upper GI communities were depleted in bacteria from the *Bacteroidetes* and *Clostridia* classes compared to lower GI, colonic contents, or stool, and enriched in *Baccili, Molicutes*, and *Gamma* and *EpsilonProteobacteria*. Several minor groups were particularly common in upper GI samples, including *Mycoplasmatales* and *Streptococaceae*. Analyses of biopsies (with adherent bacteria) from the lower GI (ascending colon) showed that they intermingle with samples of colonic contents taken at necropsy, though a distinctive feature was the abundance of *Helicobater* at this site. *Enterobacteriaceae* were far more common in the upper and lower GI samples than in stool or colonic contents, indicative of probable adherence to mucosal surfaces. Stool samples form a cluster continuous with colonic contents but extending to the upper left of the UniFrac plot. Stool samples commonly differed from colonic contents samples by having greater representation of *Spirochaetes* and several minor groups.

### Distinctive Microbial Communities Associated with Sex of the Animal of Origin

In an effort to identify additional parameters affecting the macaque GI microbiota, we asked whether communities clustered detectably in UniFrac analysis when partitioned by a variety of biological parameters. The parameters tested included sex of the animal of origin, age, disease state, antibiotic use, and viral infection. GI communities were analyzed as pools across all sample types, as pools of related samples (colonic contents plus stool), or as single sample types (stool only or colonic contents only). Unweighted UniFrac was used for these comparisons, which is based on the presence or absence of different taxa without regard to abundance.

**Figure 6:**
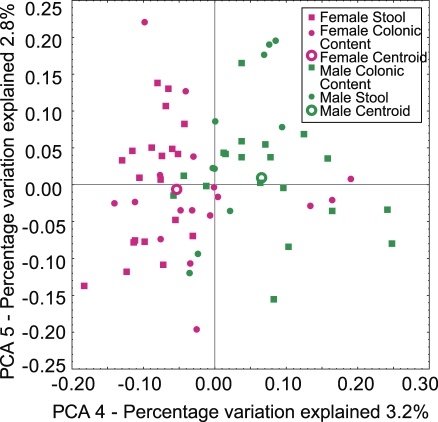
Sexual Dimorphism in the Macaque GI Microbiota Samples of stool and colonic contents are combined for this analysis. Cluster analysis was carried out using unweighted UniFrac. Separation between male communities (green) and female communities (pink) was significant (*p* < 0.05, t-test with permutation; analysis over all variation between samples). Note that with the simplest null model, we expect each Principal Coordinate to explain 100/number of samples, which is 100/100 communities = 1% of the variation. Thus the fourth Principal Coordinate, which separates males and females, is expected to contain meaningful information.

In samples of colonic contents taken at necropsy, or in samples of stool, a difference was seen between males and females. Separate clustering is illustrated for a pool of the two sample types in Figure 6 (*p* < 0.05 by t-test and label permutation). Analysis of the bacterial groups involved showed that several groups of the *Lachnospiracea* and *Bacteroidales* differed (*p* < 0.0001). One *Treponema* group was far more common in males (*p* < 0.0001). The physiological mechanism for the observed sexual dimorphism is unknown, though partitioning of the GI microbiota by sex has been noted in mice [46].

### Altered Bacterial Taxa in Animals with Colitis

**Figure 7:**
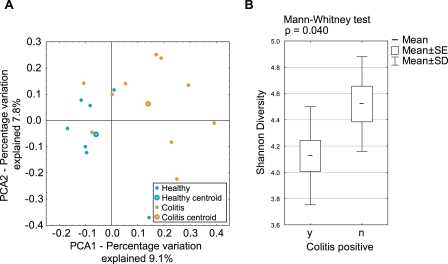
Colitis Is Associated with Distinctive GI Microbiota in Samples of Colonic Contents Taken at Necropsy The analysis was restricted to samples of colonic contents taken at necropsy that allowed unambiguous assignment to the “colitis” or “healthy” categories. (A) Analysis of communities in unweighted UniFrac. Samples in the colitis and healthy categories showed significant separation along the first principal coordinate (*p* < 0.05, t-test with permutation). For an additional four animals, insufficient clinical histories were available, so these were not included in the analysis (Table 1). (B) Diversity in samples from healthy animals or those with colitis were analyzed using the Shannon Index on OTUs condensed at 97% identity. The diversity in the samples from animals with colitis was significantly lower (*p* < 0.05; Mann-Whitney comparison of means).

The effects of disease states were then examined. Microbial communities from colonic contents were divided by whether host animals were diagnosed with colitis at necropsy (Table 1) and analyzed in unweighted UniFrac (Figure 7A). Seven samples were available for analysis from males and ten from females. Of these, nine were SIV-infected and eight were uninfected.

The communities separated along the first principal coordinate by whether the animals were diagnosed with colitis (*p* < 0.05; t-test with label permutation), indicating that the disease and associated treatment resulted in a change in composition of the GI microbiota. An analysis of the relative diversity, as reported by the Shannon Index, revealed that diversity was consistently lower in the communities from colitic animals (Figure 7B).

Most of the animals with colitis had a history of multiple bouts of diarrhea requiring medical attention including fluid therapy and in many animals treatment with antibiotics (Table S1). The antibiotics chosen for therapy differed among the animals and included tetracycline, enrofloxacin, cefazolin, and tylosin. The time of treatment relative to euthanasia and the duration of treatment also varied. Only two animals were on antibiotics (tetracycline) at the time of euthanasia. Within the cluster of communities from animals with colitis (Figure 7B), some possible sub-clustering was seen by antibiotic type, suggesting that each antibiotic resulted in characteristic changes in community composition (though larger sample sizes will be needed to assess this hypothesis definitively).

An analysis of the bacterial taxa that differed between the two groups revealed the family *Campylobacteraceae* (Epsilon-Proteobacteria) was much more common in animals with colitis–for the major *Campylobacter* OTU (97% criteria), five out of ten monkeys with colitis had this OTU, but none of the seven healthy monkeys had this OTU (G = 6.03, df = 1, *p* = 0.015). Two monkeys of unknown clinical status were also positive. A variety of additional taxa within the *Bacteroidetes* and *Firmicutes* phyla also changed in abundance significantly in assocation with colitis. The *Campylobacter* genus contains known enteric pathogens of humans ([47] and references therein), consistent with the idea that the presence of these groups was associated with pathogenesis in macaques. Of the animals detected as Campylobacter positive by sequence analysis, only two animals had positive cultures for *Campylobacter* when analyzed by conventional clinical methods.

One explanation for the enrichment of *Campylobacter* would be that antibiotic treatment created an environment favorable for colonization, as has been suggested for Clostridia difficile in humans. Of the animals with colitis that were positive for *Campylobacter*, four had histories of recent antibiotic use but three did not, and for the four treated animals three different antibiotics were used (Table S1). Thus the presence of *Campylobacter* was not strongly associated with antibiotic treatment, consistent with the idea that *Campylobacter* was associated with colitis and not antibiotic use.

SIV-infected animals were present in both the colitis and normal groups, and no strong clustering of the bacterial communities was associated with SIV infection when SIV infection was analyzed in isolation (data not shown). These data suggest that the alterations in community composition in SIV-infected animals with colitis was attributable to the colitis resulting from viral infection, and not the viral infection itself.

## Discussion

In this study, we describe the composition of 100 uncultured GI microbial communities from healthy rhesus macaques and macaques with chronic colitis. Each community was characterized by an average of ∼1,400 reads of 16S rRNA gene of median 264 nt in length. This work provides a detailed picture of the structure of the macaque GI microbiota, its dynamics, and changes associated with colitis with or without SIV infection. Macaque models are used in studying myriad GI diseases, including SIV-induced enteropathy, bacterial enteropathy, and inflammatory bowel disease. The data presented here provides detailed background, hypotheses and methods for assessing possible involvement of the full GI microbiota, and provides a model for investigating changes in the human GI microbiota in healthy and diseased individuals.

The pyrosequencing method [30] allows large numbers of 16S rRNA gene sequence reads to be obtained while controlling the costs of data acquisition, greatly increasing the number of bacterial communities and species accessible to analysis compared to culture-based methods. In the bioinformatic approach used here, the pyrosequencing reads were analyzed after first inserting them into pre-existing phylogenetic trees formed from full-length 16S rRNA gene sequences, allowing relatively accurate phylogenetic placement despite the short sequences lengths [29]. Aligning pyrosequencing reads to a pre-existing tree also serves to minimize the effects of pyrosequencing errors, since single nucleotide substitutions that cause a sequence read to align with an incorrect full length sequence will be rare.

Communities characterized by 16S rRNA gene sequence reads were compared to each other using UniFrac [38,40], which evaluates the distance between pairs of samples after alignment on phylogenetic trees based on the unique branch length leading to members of each community. One advantage of this approach is that the collection of pair-wise distances between communities can be subject to PCoA, allowing communities to be clustered along orthogonal axes of maximal variance. In a successful study of this type, clustering on each axis can report the effects of different biological variables. Previous studies of the vertebrate GI microbiota have indicated that many factors influence microbial populations, including host genotype [8,48], geography [49], antibiotic use [50], and diet [51]. Using UniFrac and PCoA, in combination with case-controlled samples, it is potentially possible to extract the effects of these and other variables and analyze each independently.

Our analysis showed that the macaque microbiota differed significantly from that of mouse or human. Even when communities from different anatomical sites were considered, or when samples from healthy hosts were mixed with diseased hosts, the effect of species of origin was still predominant. For all three vertebrates, the *Firmicutes* and *Bacteroidetes* comprised the most abundant phyla, but the composition of minor groups differed and the taxa within the *Firmicutes* and *Bacteroidetes* also differed. A distinctive feature of the macaque samples was the abundance of *Spirochaetes* from the *Treponema* lineage. These *Treponema* differ from the spiral-shaped *Helicobacter* reported previously [41], which were also detected here. Analysis of full-length 16S rRNA gene clones (Figure 1D) showed closest matches to Treponema brennaborense and Treponema saccharophilum. T. brennaborense has been associated with digital dermatitis in dairy cows [52]. T. saccharophilum has been identified as a component of the rumen GI flora that aids in digestion of pectin [53], suggesting a possible role in digesting vegetable matter in the macaque GI tract.

The analysis of healthy animals emphasized the many factors affecting composition of GI communities in macaques. The number of types of bacteria involved is very large–when macaque 16S rRNA gene sequences are grouped into OTUs at 97% or greater similarity, a threshold that has been suggested to correspond roughly to the species level, about 5,000 OTUs were identified. Microbial communities of individual animals differed from one another, and all animals followed longitudinally showed changes in community composition over time. Similarly in humans, GI microbial communities have been reported to differ among individuals and at different anatomical sites [4]. The macaque GI communities also clustered by the sex of the host animal, paralleling a proposal for sexual dimorphism in the GI microbiota in mice [46].

Samples from colonic contents of animals euthanized due to advanced colitis showed distinctive communities compared to similar samples from healthy controls, linking alterations in the GI microbial communities and GI pathogenesis. Samples from animals with colitis, whether associated with SIV infection or not, were indistinguishable. This emphasized that colitis itself (and associated therapeutic interventions) and not the cause of colitis was most tightly linked with altered GI microbiota. The presence of *Campylobacter* was strongly associated with colitis. The major *Campylobacter* OTU (97% threshold) was present in five out of ten animals with colitis, but in zero out of seven free of colitis (*p* = 0.014). Cultureable C. jejuni or C. coli were obtained only from two animals, indicating that the Campylobacter species detected were either too rare to detect by culture, or did not grow under the culture conditions used.

Most of the macaques euthanized due to GI-disease were treated with antibiotics at some point during disease progression. Thus these findings model human clinical cases where antibiotic therapy can be indicated in the treatment of colitis, but antibiotic treatment complicates analysis of effects of GI disease alone. For the samples of colonic contents taken at necropsy, there were indications of clustering due to type of antibiotic used for treatment within the larger cluster of samples from animals with colitis, though the number of samples in each antibiotic group was too low for detailed analysis by antibiotic type. Our data are consistent with the idea that the disease state caused a shift in bacterial communities that was further shaped by the antibiotics used for treatment.

The sequence-based approach described here has the potential to identify candidate pathogens involved in previously obscure disease conditions. Animal FH09 (Table S1) provides a case study. This animal suffered from prolonged chronic diarhhea of unknown cause. Exhaustive searches for a microbial pathogen by conventional culturing methods were negative. For unexplained reasons, placing the animal on a gluten-free diet helped ameliorate the condition, but eventually the animal declined and was euthanized for humanitarian reasons. Analysis of colonic contents taken at necropsy revealed a substantial number of 16S rRNA gene sequences (51 reads) that clustered with a group containing Campylobacter fetus and Campylobacter hyointestinalis. Evidently *Campylobacters* of this group are not detected in the usual culture assays. C. fetus has been implicated as an emerging pathogen and could well have been involved in the GI disease of FH09. These findings suggest that further analysis of the relationship between diet and C. fetus pathogenesis might be useful, and illustrate how the methods described here could be applied in diagnosis of human GI diseases of unknown etiology.

In summary, this study presents the first use of DNA bar coding and pyrosequencing to analyze uncultured bacterial communities from the primate gut, and provides the deepest view into the gut microbiome from the largest sample of any non-human species to date. Using the macaque model and the methods reported here, it will be possible to investigate how the interaction among bacterial community members, together with alterations in the GI environment, leads to outgrowth of pathogenic forms and resultant disease. This study also paves the way for broader application of pyrosequencing to characterize the human microbiota in health and disease, which could potentially allow large-scale characterization of thousands of human samples with orders of magnitude less expense and effort than traditional Sanger sequencing. We will thus soon be able to identify those features of the microbiota (if any) that are common to all healthy individuals, and to assess the extent to which changes in the microbiota in animal models can help guide the development of therapy for human diseases.

## Materials and Methods

### Sample collection.

Rhesus macaques (Macaca mulatta) were housed singly at the Tulane National Primate Research Center. For longitudinal studies of stool samples, four animals (CC47, FH40, CT64, DD05; here M1-M4) were infected intravenously with 100 TCID50 SIVmac251 on study day 0. Fecal samples were collected prior to infection (t1), at day 7 (t3), day 14 (t4), day 28 (t6) and day 56 (t10) post infection. These are standard time points for examination of early events in the pathogenesis of AIDS and are associated with peak viremia (day 14) and establishment of viral set point (by day 56). Stool samples for control animals (AM87, DG23, CC79, BA02; here C1-C4) were collected similarly over an eight week period. For samples of colonic contents, each was collected from the ascending colon at necropsy within one hour of euthanasia with an intravenous overdose of phenobarbital. All samples were immediately frozen to −80 °C. Samples were shipped on dry ice and stored at −80 °C until processing. In addition, intestinal biopsies of the upper (jejunum) and lower (ascending colon) were obtained by standard techniques. These biopsies were immediately frozen as for colonic contents. Housing and handling of animals were in accordance with the Guide for the Care and Use of Laboratory Animals (U.S. Public Health Service) and the Animal Welfare Act. All protocols and procedures were reviewed and approved by the Tulane University Institutional Animal Care and Use Committee. Additional animals studied, their clinical conditions, and detailed ecological descriptions of samples are in Table S1.

### Extraction and purification of DNA.

Total DNA was extracted from frozen stool using the QIAamp® DNA Stool Mini Kit (Qiagen, Inc., Valencia CA), following the manufacturer's protocol for pathogen detection.

### PCR amplification of bacterial 16S rRNA gene sequences.

For samples from each animal and at each time-point, the 16S rRNA gene was amplified from extracted DNA using the composite forward primer 5′-GCCTCCCTCGCGCCATCAGNNNN*CTGCTGCCTYCCGTA-*3′ where the underlined sequence is that of 454 Life Sciences® primer A and in italics is the broad range bacterial primer BSR357. The reverse primer was 5′-GCCTTGCCAGCCCGCTCAGNNNN *AGAGTTTGATCCTGGCTCAG*-′3, where the underlined sequence is that of 454 Life Sciences® primer B and in italics is the broad range bacterial primer BSF8. The NNNN designates the unique four base bar code used to tag each PCR product. Reaction conditions were as follows: 5.0 μl 10× PCR buffer II (Applied Biosystems, Foster City, CA), 3.0 μl MgCl2 (25 mM; Applied Biosystems), 2.5 μl Triton X-100 (1%), 2.0 μl deoxyribonucleoside triphosphates (10 mM), 1.0 μl forward primer and 1.0 μl reverse primer (20 pmol/μl each) and 0.5 μl AmpliTaq® DNA polymerase (5U/μl; Applied Biosystems) and 100 ng of template DNA in a total reaction volume of 50 μl. Reactions were run in a GeneAmp® PCR System 9700 cycler (Applied Biosystems) using the following cycling parameters: 5 minutes denaturing at 95 °C followed by 20 cycles of 30 secs at 95 °C (denaturing), 30 secs at 56 °C (annealing) and 90 secs at 72 °C (elongation), with a final extension at 72 °C for 7 minutes. Four independent PCR reactions were performed for each sample along with a no template negative control.

### Gel purification and pyrosequencing.

Each PCR product was gel purified from a 0.8% agarose gel. DNA was isolated using the QIAquick® Gel extraction kit (Qiagen, Inc., Valencia CA). 100 ng of each of the 100 gel purified DNAs was added to a master pool of DNA which was sent for pyrosequencing with primer A as described [30,54]. Several studies have analyzed sources of error in 454 sequencing runs, which informed our choices for quality control here [37,54,55]. For a sequence to pass quality control, it needed to (1) show a perfect match to the bar code and 16S rRNA gene primer, (2) be at least 50 nt in length, (3) have no more than two undetermined bases in the sequence read, and (4) find at least a 75% match to a previously determined 16S rRNA gene sequence after alignment with NAST (http://greengenes.lbl.gov/). The sequences were inserted into the 16S rRNA gene tree constructed by Hugenholz et al. [56] using the parsimony insertion tool from ARB software (http://www.arb-home.de/). A “termini” filter was used for the parsimony insertion. After applying this criteria, 36,652,141 bases of sequence were available for analysis. All sequence data will be deposited at NCBI upon acceptance of this manuscript for publication.

### Bioinformatic analysis.

OTU clustering and analysis was carried out using OTUPicker (M. Hamady and R. Knight, unpublished). Clustering and principal coordinate analysis were conducted using UniFrac [29,38,39]. UniFrac analysis can be carried out based on the presence and absence of bacterial taxa (unweighted UniFrac), or taking into account abundance information on each group (weighted UniFrac); Figures 3, 4, 6, and 7 report unweighted UniFrac results. To perform permutation tests within UniFrac, we randomized the labels of each group and repeated the cluster analysis. We then compared all distances between points that both came from the same group to all distances between points that came from different groups using a t-test. In the permutation test, we obtained a nonparametric distribution for the t statistic that takes into account the correlations introduced by the distance matrix structure. We used 1,000 permutations, so we cannot specify *p*-value more precisely than “<0.001” if none of the permuted sets gave a more extreme result than the actual set. We note that the principal coordinate analysis assumes that the relationships between taxon abundance and environmental gradients is linear. In choosing the Monte Carlo methods used for significance testing, we accepted reduced power to avoid using parametric methods, which assume random distribution in the error terms. The taxonomy assignments were based on the group names in Arb. Ecological parameters in Table S1 were calculated using OTUPicker and PAST [57].

Errors in pyrosequencing may occur at a rate of about 0.25% [37], suggesting that the most of the 260-nucleotide sequences that remain after filtering will contain either 0 or 1 errors. Single- nucleotide errors will not affect either of the analyses we present (high-level taxonomic breakdowns or UniFrac) substantially, as they are unlikely to cause assignment of pyrosequence reads to the wrong taxonomic group and contribute almost no branch length to the phylogenetic tree used for UniFrac analyses. However, these sequencing errors could affect estimates of the total number of OTUs at a given threshold, so some caution in interpreting the total number of species-level taxa in the samples is required. Using the Poisson model, we would expect only 4.4 × 10−5% of the reads to contain the seven errors that would be required to form a new species-level at the 97% OTU threshold. Thus, it is unlikely that a single OTU in the analysis was generated through that mechanism.

## Supporting Information

Table S1 Characteristics of Samples of Uncultured Macaque GI Communities Used in This Study This table provides the description of monkeys sampled, clinical parameters for disease states, and ecological statistics describing the communities sampled.
(93 KB XLS)

Table S2 Near-full-length 16S rRNA Gene Sequences Determined by the Sanger Method, and Their Taxonomic Positions These sequences allow finer discrimination of the major macaque GI bacterial taxa.
(404 KB XLS)

## References

[ppat-0040020-b001] Ley RE, Peterson DA, Gordon JI (2006). Ecological and evolutionary forces shaping microbial diversity in the human intestine. Cell.

[ppat-0040020-b002] Whitman WB, Coleman DC, Wiebe WJ (1998). Prokaryotes: the unseen majority. Proc Natl Acad Sci U S A.

[ppat-0040020-b003] Gill SR, Pop M, Deboy RT, Eckburg PB, Turnbaugh PJ (2006). Metagenomic analysis of the human distal gut microbiome. Science.

[ppat-0040020-b004] Eckburg PB, Bik EM, Bernstein CN, Purdom E, Dethlefsen L (2005). Diversity of the human intestinal microbial flora. Science.

[ppat-0040020-b005] Palmer C, Bik EM, Eisen MB, Eckburg PB, Sana TR (2006). Rapid quantitative profiling of complex microbial populations. Nucleic Acids Res.

[ppat-0040020-b006] Palmer C, Bik EM, Digiulio DB, Relman DA, Brown PO (2007). Development of the human infant intestinal microbiota. PLoS Biol.

[ppat-0040020-b007] Turnbaugh PJ, Ley RE, Mahowald MA, Magrini V, Mardis ER (2006). An obesity-associated gut microbiome with increased capacity for energy harvest. Nature.

[ppat-0040020-b008] Ley RE, Backhed F, Turnbaugh P, Lozupone CA, Knight RD (2005). Obesity alters gut microbial ecology. Proc Natl Acad Sci U S A.

[ppat-0040020-b009] Scanlan PD, Shanahan F, O'Mahony C, Marchesi JR (2006). Culture-independent analyses of temporal variation of the dominant fecal microbiota and targeted bacterial subgroups in crohn's disease. J Clin Microbiol.

[ppat-0040020-b010] Manichanh C, Rigottier-Gois L, Bonnaud E, Gloux K, Pelletier E (2006). Reduced diversity of faecal microbiota in crohn's disease revealed by a metagenomic approach. Gut.

[ppat-0040020-b011] Fagarasan S, Muramatsu M, Suzuki K, Nagaoka H, Hiai H (2002). Critical roles of activation-induced cytidine deaminase in the homeostasis of gut flora. Science.

[ppat-0040020-b012] Suzuki K, Meek B, Doi Y, Muramatsu M, Chiba T (2004). Aberrant expansion of segmented filamentous bacteria in IgA-deficient gut. Proc Natl Acad Sci U S A.

[ppat-0040020-b013] Ayabe T, Satchell DP, Wilson CL, Parks WC, Selsted ME (2000). Secretion of microbicidal alpha-defensins by intestinal paneth cells in response to bacteria. Nat Immunol.

[ppat-0040020-b014] Hooper LV, Stappenbeck TS, Hong CV, Gordon JI (2003). Angiogenins: a new class of microbicidal proteins involved in innate immunity. Nat Immunol.

[ppat-0040020-b015] Li Q, Duan L, Estes JD, Ma ZM, Rourke T (2005). Peak SIV replication in resting memory CD4+ T cells depletes gut lamina propria CD4+ T cells. Nature.

[ppat-0040020-b016] Veazey RS, DeMaria M, Chalifoux LV, Shvetz DE, Pauley DR (1998). Gastrointestinal tract as a major site of CD4+ T cell depletion and viral replication in SIV infection. Science.

[ppat-0040020-b017] Mehandru S, Poles MA, Tenner-Racz K, Horowitz A, Hurley A (2004). Primary HIV-1 infection is associated with preferential depletion of CD4+ T lymphocytes from effector sites in the gastrointestinal tract. J Exp Med.

[ppat-0040020-b018] Guadalupe M, Reay E, Sankaran S, Prindiville T, Flamm J (2003). Severe CD4+ T-cell depletion in gut lymphoid tissue during primary human immunodeficiency virus type 1 infection and substantial delay in restoration following highly active antiretroviral therapy. J Virol.

[ppat-0040020-b019] Guadalupe M, Sankaran S, George MD, Reay E, Verhoeven D (2006). Viral suppression and immune restoration in the gastrointestinal mucosa of human immunodeficiency virus type 1-infected patients initiating therapy during primary or chronic infection. J Virol.

[ppat-0040020-b020] Brenchley JM, Price DA, Schacker TW, Asher TE, Silvestri G (2006). Microbial translocation is a cause of systemic immune activation in chronic HIV infection. Nat Med.

[ppat-0040020-b021] Knox TA, Spiegelman D, Skinner SC, Gorbach S (2000). Diarrhea and abnormalities of gastrointestinal function in a cohort of men and women with HIV infection. Am J Gastroenterol.

[ppat-0040020-b022] Heise C, Miller CJ, Lackner A, Dandekar S (1994). Primary acute simian immunodeficiency virus infection of intestinal lymphoid tissue is associated with gastrointestinal dysfunction. J Infect Dis.

[ppat-0040020-b023] Stone JD, Heise CC, Miller CJ, Halsted CH, Dandekar S (1994). Development of malabsorption and nutritional complications in simian immunodeficiency virus-infected rhesus macaques. Aids.

[ppat-0040020-b024] Mohan M, Aye PP, Borda JT, Alvarez XJ, Lackner AA (2007). Gastrointestinal disease in SIV-infected rhesus macaques is characterized by proinflammatory dysregulation of the IL-6-JAK-STAT3 pathway. Am J Path.

[ppat-0040020-b025] Sestak K, Merritt CK, Borda J, Saylor E, Schwamberger SR (2003). Infectious agent and immune response characteristics of chronic enterocolitis in captive rhesus macaques. Infect Immun.

[ppat-0040020-b026] Ramesh G, Alvarez X, Borda JT, Aye PP, Lackner AA (2005). Visualizing cytokine-secreting cells in situ in the rhesus macaque model of chronic gut inflammation. Clin Diagn Lab Immunol.

[ppat-0040020-b027] De Hertogh G, Aerssens J, de Hoogt R, Peeters P, Verhasselt P (2006). Validation of 16S rDNA sequencing in microdissected bowel biopsies from crohn's disease patients to assess bacterial flora diversity. J Pathol.

[ppat-0040020-b028] Pace NR, Olsen GJ, Woese CR (1986). Ribosomal RNA phylogeny and the primary lines of evolutionary descent. Cell.

[ppat-0040020-b029] Liu Z, Lozupone C, Hamady M, Bushman FD, Knight R (2007). Short pyrosequencing reads suffice for accurate microbial community analysis. Nucleic Acids Res.

[ppat-0040020-b030] Margulies M, Egholm M, Altman WE, Attiya S, Bader JS (2005). Genome sequencing in microfabricated high-density picolitre reactors. Nature.

[ppat-0040020-b031] Dedysh SN, Pankratov TA, Belova SE, Kulichevskaya IS, Liesack W (2006). Phylogenetic analysis and in situ identification of bacteria community composition in an acidic sphagnum peat bog. Appl Environ Microbiol.

[ppat-0040020-b032] Schmitt-Wagner D, Friedrich MW, Wagner B, Brune A (2003). Phylogenetic diversity, abundance, and axial distribution of bacteria in the intestinal tract of two soil-feeding termites (cubitermes spp.). Appl Environ Microbiol.

[ppat-0040020-b033] Salzman NH, de Jong H, Paterson Y, Harmsen HJ, Welling GW (2002). Analysis of 16S libraries of mouse gastrointestinal microflora reveals a large new group of mouse intestinal bacteria. Microbiology.

[ppat-0040020-b034] Ludwig W (2004). ARB: a software environment for sequence data. Nucleic Acids Res.

[ppat-0040020-b035] DeSantis TZ, Hugenholtz P, Keller K, Brodie EL, Larsen N (2006). NAST: a multiple sequence alignment server for comparative analysis of 16S rRNA genes. Nucleic Acids Res.

[ppat-0040020-b036] Stackebrandt E, Goebal B (1994). Taxonomic note: a place for DNA-DNA reassociation and 16s rRNA sequence analysis in the present species definition in bacteriology. Int Syst Bacteriol.

[ppat-0040020-b037] Huse SM, Huber JA, Morrison HG, Sogin ML, Welch DM (2007). Accuracy and quality of massively parallel DNA pyrosequencing. Genome Biol.

[ppat-0040020-b038] Lozupone C, Hamady M, Knight R (2006). UniFrac–an online tool for comparing microbial community diversity in a phylogenetic context. BMC Bioinformatics.

[ppat-0040020-b039] Lozupone CA, Hamady M, Kelley ST, Knight R (2007). Quantitative and qualitative (beta} diversity measures lead to different insights into factors that structure microbial communities. Appl Environ Microbiol.

[ppat-0040020-b040] Lozupone C, Knight R (2005). UniFrac: a new phylogenetic method for comparing microbial communities. Appl Environ Microbiol.

[ppat-0040020-b041] Duhamel GE, Stryker CJ, Lu G, Wong VJ, Tarara RP (2003). Colonic spirochetosis of colony-raised rhesus macaques associated with brachyspira and helicobacter. Anaerobe.

[ppat-0040020-b042] Duhamel GE, Elder RO, Muniappa N, Mathiesen MR, Wong VJ (1997). Colonic spirochetal infections in nonhuman primates that were associated with brachyspira aalborgi, serpulina pilosicoli, and unclassified flagellated bacteria. Clin Infect Dis.

[ppat-0040020-b043] Munshi MA, Taylor NM, Mikosza AS, Spencer PB, Hampson DJ (2003). Detection by PCR and isolation assays of the anaerobic intestinal spirochete brachyspira aalborgi from the feces of captive nonhuman primates. J Clin Microbiol.

[ppat-0040020-b044] Xu J, Bjursell MK, Himrod J, Deng S, Carmichael LK (2003). A genomic view of the human-bacteriodetes thetaiotaomicron symbiosis. Science.

[ppat-0040020-b045] Xu J, Mahowald MA, Ley RE, Lozupone CA, Hamady M (2007). Evolution of symbiotic bacteria in the distal human intestine. PLoS Biol.

[ppat-0040020-b046] Schloss PD, Handelsman J (2006). Introducing SONS, a tool for operational taxonomic unit-based comparisons of microbial community memberships and structures. Appl Environ Microbiol.

[ppat-0040020-b047] Parkhill J (2000). The genome sequence of the food-borne pathogen campylobacter jejuni reveals hypervariable sequences. Nature.

[ppat-0040020-b048] Zoetendal EG, Akkermans AD, Akkermansvan Vliet WM, De Visser AGM, De Vos WM (2001). The host genotype affects the bacterial community in the human gastrointestinal tract. Microbial Ecol Health Dis.

[ppat-0040020-b049] Lay C, Rigottier-Gois L, Holmstrom K, Rajilic M, Vaughan EE (2005). Colonic microbiota signatures across five northern european countries. Appl Environ Microbiol.

[ppat-0040020-b050] Young VB, Schmidt TM (2004). Antibiotic-associated diarrhea accompanied by large-scale alterations in the composition of the fecal microbiota. J Clin Microbiol.

[ppat-0040020-b051] Tannock GW, Munro K, Bibiloni R, Simon MA, Hargreaves P (2004). Impact of consumption of oligosaccharide-containing biscuits on the fecal microbiota of humans. Appl Environ Microbiol.

[ppat-0040020-b052] Dermirkan I, Williams HF, Dhawi A, Carter SD, Winstanley C (2006). Characterization of a spirochaete isolated from a case of bovine digital dermatitis. J Appl Microbiol.

[ppat-0040020-b053] Paster BJ, Canale-Parola E (1985). Treponema saccharophilum sp. nov., a large pectinolytic spirochete from the bovine rumen. Appl Environ Microbiol.

[ppat-0040020-b054] Sogin ML, Morrison HG, Huber JA, Welch DM, Huse SM (2006). Microbial diversity in the deep sea and the underexplored “rare biosphere”. Proc Natl Acad Sci U S A.

[ppat-0040020-b055] Hoffmann C, Minkah N, Leipzig J, Wang G, Arens MQ (2007). DNA bar coding and pyrosequencing to identify rare HIV drug resistance mutations. Nucleic Acids Res.

[ppat-0040020-b056] Hugenholtz P (2002). Exploring prokaryotic diversity in the genomic era. Genome Biol.

[ppat-0040020-b057] Hammer O, Harper DAT, Ryan PD (2001). PAST: paleontological statistics software package for education and data analysis. Paleontologica Electronica.

